# Developmental diversity: Putting the development back into research about developmental conditions

**DOI:** 10.3389/fpsyt.2022.986732

**Published:** 2023-01-06

**Authors:** Kristien Hens, Leni Van Goidsenhoven

**Affiliations:** Department of Philosophy, University of Antwerp, Antwerp, Belgium

**Keywords:** autism, ethics, development, diversity, humanities and social science, neurodiversity

## Abstract

The dominant discourse surrounding neurodevelopmental conditions such as autism and ADHD emphasizes biological explanations. Neurodevelopmental conditions are conceived as different types of brains, the result of different types of genes. This way of thinking is present both in medical research and in clinical practice. Indeed, it is widely acknowledged that the idea of having a biological diagnosis helps people see beyond blame and guilt. It aids acceptance. However, simplistic approaches to biology risks neglecting the experiences and stories of autistic people in favor of finding etiological causes. At the same time, there is growing awareness that risks, functioning, and resilience are not solely defined by genes and brains but have a cultural and experiential component as well. Furthermore, atypical cognitive trajectories are not straightforwardly associated with poor outcomes. In this paper we describe the concept of developmental diversity as an alternative to more categorical approaches to neurodevelopmental conditions. We explore how dynamic models of life offer possibilities to look at neurodevelopmental conditions differently: rather than seeing autistic people as people with fundamental flaws in their genes or software faults in their brains that have to be explained, autism appears as a phenomenon that exists in interaction with the context, as a meaningful reaction to the environment. We explore what it would mean for research to go from a diagnosis-based approach to a developmental diversity approach that will define wellbeing and functioning in a more granular way across developmental trajectories. We argue that this would mean incorporating lived experiences into biological research and going beyond genes-environment dichotomies. Next to yielding a more complete picture on the phenomenon of autism, we describe how an approach that takes developmental diversity as a starting point offers a new way to look at existing challenges of autism research, such as how to deal with the significant overlap between diagnosis. Our hypothesis is that thinking with developmental diversity rather than categorical difference both represents an opportunity for a more inclusive society, and fundamentally can alter the way we perform research. As such, it is in line with requests of neurodiversity and disability movements.

## Introduction: Autism and biology

The dominant discourse surrounding neurodevelopmental disabilities, such as autism and ADHD, emphasizes straightforward biological explanations. Neurodevelopmental disabilities are conceived as different types of brains resulting from different genes. In the case of autism, often, explanatory models are presented as composed of different layers, influencing each other downstream. In autism research, clinical practice and the general public, it is accepted that genes cause brain differences, which cause different modes of cognitive functioning. Such cognitive functioning is then reflected in behavior, which in its turn forms the basis of formal diagnosis, done through behavioral assessment and assessment of functioning (1). At the same time, autism is considered heterogeneous, meaning different people can exhibit other behaviors more or less (2).^1^ This heterogeneity is not only present at the level of the behavior but also at the level of the genes (3). Nevertheless, after decades of genetic research on autism, the one conclusion that researchers have drawn is that the idea of a “gene for autism” should be given up. Many different genes seem to play a role, and the genes associated with autism are also associated with other conditions such as ADHD—as the adage goes: genes do not think in DSM terms (4).^2^ At the same time, *heterogeneity* suggests that there is at least a factor that binds different manifestations together.

There have been various explanatory models for autistic behavior (7, 8). For example, some older models, such as deficit in Theory of Mind, focused on the social and communicative atypicalities in the behavior of autistic people. These models explained autism primarily as a social deficit (9). Other models focus on differences in information processing, such as the Enhanced Perceptual Functioning hypothesis or the High Inflexible Precision of Prediction Errors (HIPPEA) hypothesis (10, 11), or increased sensory perception (12). These explanatory models are not easily reduced to one another: it is one thing to say that specific autistic behavior that some would call “socially awkward” is due to a lack of social insight or of “theory of mind,” and it is another thing to state that it is due to the effort it takes for autistic people to deal with incoming sensory and informational stimuli. There is also a fundamental difference between claiming, for instance, that autistic people have atypical eye contact because they do not understand that the eyes are mirrors of the soul or do not understand that other people have such things as minds and saying that autistic people have atypical eye contact because other people's gaze is too intense, borderline insupportable (13). As we have argued elsewhere, the explanatory model one chooses is not without its therapeutic and normative consequences (1, 14, 15). If one thinks autism is due to a deficit of social cognition, therapy will focus on teaching social skills and scripts rather than avoiding too intense stimuli. For research as well, this has far-reaching implications, as one's idea about autism will guide the choice of experiments and brain regions to investigate.

Nevertheless, despite this heterogeneity, these genetic and cognitive explanatory models of autism suggest that autism is a relatively stable given (2, 16). The idea that there is a stable core to autism is not only present in research. Also in clinical contexts, it is often assumed that we can delineate and define autism and that it has an essence that we can pin down. The underlying idea is that it may be so that we do not wholly understand what autism is at the moment, but we do know that it has a biological underpinning that can be discovered. This assumption has, of course, several implications. First, it is widely acknowledged that the idea of having a precise biological diagnosis helps people see beyond blame and guilt (17): it aids acceptance and offers parents of autistic children and autistic people themselves a handhold, a name with which to identify (18). However, the role that this aspect of biological certainty plays in (self-)acceptance and the factual lack of that biological certainty puts clinicians and diagnosticians in a dilemma (19–21). For instance, in informal conversations with the authors of this paper, they often acknowledge that little is known about the autistic brain or the brain in general. Still, they admit that presenting autism as a different kind of brain, with scientific and biological certainty, is helpful for people in diagnostic processes.

This sentiment (the idea of using the narrative of a different kind of brain, biological certainty and cause clarity) keeps explanatory research into the causes of autism very much alive. Clinicians still often express their hope for biomarkers that would help diagnose autism with biological certainty. They feel that this would give some confidence and some gist to what is now a diagnosis based on behavior. Also, autistic people themselves often welcome this certainty (18). As such, a *raison-d-être* is provided to the search for (somewhat reductionist) biological explanations for autism (20, 22). At the same time, we may wonder what biology's unique role is in providing such certainty.

Approaches to autism that start from a reductionist view on biology, for example, because they claim that autism is straightforwardly caused by “genes” or is a “differently wired brain” risk neglecting, among others, the experiences and stories of autistic people in favor of finding etiological causes. It also does not sufficiently engage with the neurodiversity-affirmative paradigm—which is increasingly acknowledged as relevant for autism research (23–25). We, therefore, assert that the idea that “genes cause behaviour” is naive at best and dangerous at worst. In line with that, we notice a growing awareness that risks, functioning, and resilience are not solely defined by genes and brains but are situated and thus have a cultural and experiential component (26, 27). Furthermore, atypical cognitive trajectories are not straightforwardly associated with poor outcomes in terms of wellbeing (28, 29). Integrating this knowledge and insights into new research and the autism discourse is essential (30). Nevertheless, we do not suggest that biological approaches to autism are wrong *per se* or that research into the biological underpinnings of autism is not interesting anymore. Instead, we are critical of reductionist approaches to biology. We want to point out that research incorporating systemic approaches (also called integrative approaches) to biology, and thus incorporating culture, experience, dynamics and development, will benefit autistic people, their kin and autism science in general. *In this article, we suggest that developmental diversity as the starting point for research, rather than categorical diagnosis, helps conceptualize what such research might entail*. We proceed as follows: we will first describe the concept of neurodiversity and its relation to developmental diversity, stressing that neither term aims to romanticize autism or minimize challenges that people with diagnoses may encounter. We then situate the concept of development in philosophy and the history of science and autism. We end with giving some suggestions as to what a developmental diversity approach in autism research could entail.

## Developmental diversity and neurodiversity

*Developmental diversity* is, of course, not a new term. Like the term “neurodiversity,” it is sometimes used in project proposals and the clinic as an alternative to “developmental disorder.” Both terms, then, convey that autism and other developmental conditions (e.g., ADHD, Tourette, …) should not be seen as a “disorder” or a “disease” but rather as a human difference. The central premise of both terms is that diversity in development and functioning across humans is “a natural and valuable part of human variation” (31). However, it must be said that while developmental diversity and neurodiversity are complementary, they are not synonyms. It is essential to focus first on what *neurodiversity and neurodiversity-affirmative autism research* mean to understand what we put forward with the notion *of developmental diversity*.

Neurodiversity has its value as a political term referring to justice in the context of developmental disabilities. When we engage with the history of the neurodiversity movement, we notice that in the early 1990s, neurodiversity was primarily connected to identity politics. The notion emerged mainly in English-speaking online communities of autistic individuals and pointed out that autism is not something to be cured but is a natural part of diversity across humans. This acknowledgment does not imply that autism is not understood as a *disability*. Indeed, within the neurodiversity movement, autism is conceptualized using the social model of disability (32). This means that disability is conceptualized as resulting from a poor fit between a given individual's (physical, cognitive or emotional) characteristics and the characteristics of their social context. A disability is not simply a defect in the individual. It arises from the interaction between a person and an unaccommodating environment (23, 31). Even for those with the highest support needs, disability can often be minimized or avoided through environmental change and the provision of appropriate assistive tools. For instance, providing a non-speaking or minimal-verbal autistic person with an alternative method of communication may give them a voice (33), but, as den Houting states: “they will only truly stop being disabled when others listen” (23).

So, drawing on the social model of disability, neurodiversity was thus initially mainly deployed as a socio-political identity in line with other minority groups. According to this perspective, autistic people could (perhaps for the first time) be proud of their autism and claim political rights to promote social participation. But as with any social justice movement, this neurodiversity movement is not without its critiques (23). For instance, some stakeholders—mainly parents of autistic children with substantial intellectual, language and behavioral challenges—argue (d) that the neurodiversity movement (primarily consisting of verbal autistic adults without these challenges) does not represent their children's experience and that their children require interventions to achieve a reasonable quality of life (25, 31, 34). Although this is quite a challenging disagreement that needs more participatory action-research^3^, it is essential to emphasize that the neurodiversity movement is not categorically opposed to support or intervention, as we will explain in more depth below.

Over the years, neurodiversity as a movement became supplemented by neurodiversity as a *standpoint*, indicating a critical attitude toward the frameworks on which our thinking and value systems are founded (24, 39, 40). Related, neurodiversity has also been conceptualized as a *new paradigm*, one that challenges the dominant paradigm that considers autism and other neurotypes as problems to be cured or solved (41). As a *standpoint*, neurodiversity deconstructs the neurotypical as a norm; it points out that also the dominant frames of thought and value systems are not self-evident or natural but have gained authority through particular contexts. This deconstruction and critical attitude is much needed because research and practice have been failing autistic people of all kinds for decades, promoting models that stigmatize more than they support (25). Moreover, all of this happened mainly without the input of any autistic people at all (42). So, taking a neurodiversity stance means a shift in focus from pathology toward neurodivergent wellbeing and lived experiences, as well as the inclusion and leadership of autistic people. A neurodivergent standpoint challenges the imaginary ideal of a cognitively “normal” subject and dominant notions of being human. It will also foreground complexity and ambiguity and multiple ways of being literate or social rather than working with clear structural barriers of normality that exclude people, as much is lost in reduction. As Erin Manning, a philosopher working on neurodiversity, argues: “Ambiguity is actually something to be embraced rather than to be avoided. It is an inevitable feature of human discourse” (43). It may be evident by this that a neurodiversity standpoint is not a synonym for an “autistic perspective,” just as neurotypical does not simply stand for “non-autistic.” Instead, the neurotypical standpoint stands for the dominant and, at the same time, invisible, so-called “neutral” stance that determines how we view concepts such as normality, knowledge, communication, a good life, etc. For example, a neurotypical view highly values rational learning, cognition, and independence. In this view, Intuition, dependence, and loving care are mostly not seen as full-fledged sources of knowledge (44). A neurodiversity standpoint instead raises critical questions about this. It deconstructs a society based on mental/neurological normality and autonomy and seeks to appreciate complex forms of dependency and otherness. It questions who determines what knowledge is and how it is valued—it stresses that science is never value-free (45).

Acknowledging the importance of including many different voices and appreciating different ways of being human does not mean that a neurodiversity stance opposes clinical support or intervention. Nor does it want to deprioritize medical research, block clinical care or neglect the difficulties an autistic person can experience.^4^ Quite the contrary: neurodiversity stresses the equal value of every human being, promotes autistic rights (and these rights can include intervention and support whenever needed), de-stigmatizes autism and creates space for epistemic justice in conceptualizing health, disability and what it means to be human (46, 47).

The growth of the neurodiversity stance has brought about new ethical, theoretical, and political debates within autism theory, research and practice during the last 5 years. Some argue that autism research is structurally changing from “normal science” to participatory neurodiversity-affirmative autism science (31, 48, 49). Thus, autism research is gradually embracing the *neurodiversity paradigm*. It may be tempting to think that the shift from pathology to neurodivergent wellbeing and lived experiences mainly impacts autism research focused on adults.^5^ However, more recently, there is increasingly more research into the implications of a neurodiversity-affirmative framework for early detection, interventions and therapy (31, 34, 50, 51). Of course, as Sue Fletcher-Watson points out, neurodiversity-affirmative early interventions research for children (with and without more profound intellectual, language and behavioral challenges) has several implications:

*As researchers and practitioners, we need to be prepared to throw away the text book on what we think we know about early development. This includes radically re-thinking our language. I've used terms “intervention” and “outcome” here on purpose in order to highlight the contradictions, but increasingly I am learning to think about this topic in terms of support, growth and wellbeing. We must ask ourselves, what are the truly important outcomes and reasonable routes to those outcomes? And in doing so we need to incorporate diverse perspectives from the autism community* *(**34**)*.

Leadbitter et al. reflect upon this in their study on neurodiversity-affirmative early intervention:

*Whilst diversity brings fundamental collective advantages, within any one neurodivergent individual weaknesses are often the inextricable partner of strengths, and that individuals can want things to be different and still want to be themselves. It includes the understanding that some neurological differences are disadvantageous, either inherently or in interaction with the environment, and could benefit from correspondingly targeted intervention* *(**31**)*.

In other words, when scientists challenge normative thinking about (early) development and when early interventions aim to provide opportunities for physical, sensory and emotional regulation, they can be compatible with the neurodiversity stance.

Important in the context of this article is also the connection of neurodiversity with biology. As the neurodiversity standpoint is not opposed to clinical intervention and support, it is not opposed to biological research about developmental conditions either. However, it does react against biological essentialism and the comparatively individualistic, fitness-based evolutionary model. Often neurodiversity scholars such as Robert Chapman assume an ecological model influenced by how ecologists talk about functioning (24). Chapman describes how ecologists are less interested in ranking individual fitness levels. They investigate how broader systems function as a whole, how functions emerge from relations between organisms, and how the dominance of some forms of organisms can be harmful to the functioning of others (24, 47, 52).^6^

Neurodiversity-affirmative autism research and clinical practice is the right way toward ethical and just research and practice. We argue that all autism research and intervention stakeholders must actively form partnerships with autistic people and engage with and understand neurodiversity as a concept, standpoint and movement. In so doing, we move away from both a deficit and individualist model and the idea that “normality” is what we should aim for. Such an approach implies reframing effectiveness, paying attention to environmental goodness-of-fit, developing tools to measure autistic prioritized outcomes, internal drivers and experiences, and focusing on autistic prioritized intervention targets (31). In this way, autistic developmental trajectories are taken seriously. Here neurodiversity connects with developmental diversity. Neurodiversity is *why* and *how* developmental diversity should be studied. Developmental diversity, as the object of neurodevelopmental research, embraces the neurodiversity idea *that developmental differences are always to be understood in relation to context and specific moments in time and beyond categorical boundaries*. To truly grasp a phenomenon such as autism, it is hence not only essential to explain it by referring to biological underpinnings. Such explaining can only ever be truthful if it is inspired by an understanding of what certain behaviors and experiences actually mean for a person. Before discussing how such research could be done, we will first try to understand what “development” means.

## Dynamics and development: Systems biology and its conundrums

In the DSM-5, some explanation is given as to why conditions such as autism, ADHD and Tourette's are called *neurodevelopmental*. Unlike other diagnoses that are defined in the DSM, which often occur in or after adolescence, neurodevelopmental conditions are those conditions that start at an early point in life (5). It is deliberately left vague what this early period is and whether or when it ends, develops or changes. Moreover, not much is said about the causes of such developmental disorders. It is not because the first symptoms of autism occur during the first years of childhood that autism is caused by something that happened during those first years. The conceptualization of autism as a developmental disorder is also reflected in the diagnostic criteria of autism itself. Besides the wellknown behavioral criteria, the DSM-5 states that “Symptoms must be present in the early developmental period (but may not become fully manifest until social demands exceed limited capacities or may be masked by learned strategies in later life)” (5). Hence, people may be diagnosed later in life, but there must be proof that symptoms were already there in early childhood, although they may not have led to dysfunction. This requirement seeks to distinguish so-called “real autism” from, for example, conditions that may be associated with the same symptoms but that may result from trauma or other events that happened later in development (53, 54). Indeed, given the history of autism and the harmful “mother blaming” discourse of the second half of the twentieth century, much is at stake when we think about the origins of autism, and the suggestion that autism might be caused by psychosocial deprivation is contentious. For example, there seems to be a tendency to distinguish between “true autism” and “quasi-autism.” The first one, “true autism,” would then be the kind with which one is born as it is genetic (and so, for which no one is to blame). At the same time, “quasi-autism” refers to young children who show autistic-like patterns but where it is assumed that some adverse experience causes the behavior. Hence, the cause of their quasi-autism is supposedly genetic, but rather psychological deprivation as they, for instance, were reared in profoundly depriving institutions, such as some Romanian orphanages in the 1970 and 80s (53).

As stated above, “developmental,” when referring to developmental disorders as defined in the DSM-5, refers to the manifestation of the behavior in the early years. It is assumed that autism is present from birth as a genetic variant that starts manifesting in the period of life where the behavior at stake becomes relevant. By equating development thus with the period of manifestation, a static concept of autism as an innate neuroatypicality is safeguarded. However, development also implies dynamics: an unfolding of form in reaction to internal genetic “programming,” life events and environment. In what follows, we will first sketch a general discussion of development from a philosophy and history of science perspective. Secondly, we will show that the tension between static, dynamic and developmental views on autism has existed from the beginning. Finally, we will state that this tension is also visible in the characteristics of current-day autism research, which often centers around genes, early detection and early intervention.

### Development from a philosophy and history of science perspective

When diving into the discussions surrounding development from a philosophy and history of science perspective, we notice that the concept of “development” ties in with centuries-old discussions about the origins of forms (55). It is related to the debate on epigenesis vs. preformation. Epigenesis, in this sense, is a view of the development of organisms and is contrasted with preformation. A preformationist theory assumes that an organism's eventual form is already there from conception onwards. Think about the seventeenth-century idea of the homunculus. After discovering gametes, some researchers then assumed that the sperm cell would contain a “little man,” which would merely become enlarged during development.

It has been suggested that the idea that what organisms will become is more or less fixed in the combination of genes acquired upon the fusion of the sperm and the genes can be characterized as somewhat preformationist. In the mid-twentieth century, Conrad Waddington introduced the idea of the epigenetic landscape (56). Waddington used the image of the landscape with valleys and hills to describe the development of a phenotype. Every cell has the same nuclear DNA, but they develop into specific types of cells depending on the place in the organism. Waddington describes two crucial concepts: plasticity and canalization. Plasticity is the ability of a given genotype to give rise to different types of cells in response to environmental circumstances, such as the place in the organism (56–59). Canalization is the adjustment of the developmental pathways to bring about a uniform developmental result despite genetic and environmental variations. For Waddington, it is not the genes that influence the landscape but a network of genes. Because of the canalization, a minor rearrangement will not significantly affect the cells' trajectories. However, if the landscape is wholly rearranged because of changes in the underlying network of genes or environmental changes, this will severely impact development. It is important to note that canalization and plasticity are not each other's opposites. They imply each other. Canalized development requires some plasticity to adapt to different circumstances (60).

Furthermore, adapting to different circumstances implies stability to withstand total annihilation. Indeed, stability requires dynamics to keep systems stable. Recently, Developmental Systems thinkers, inspired by Waddington and others, have challenged the predominance of the gene in thinking about organisms (61, 62). They do not want to deny the relative importance of genes in development, nor are they environmentalists in that they shift the balance toward the environment. Instead, they argue against a dualistic interpretation of causes as either genes or environment. Genes and many other factors play a role in life, and myriad interactions and interplays are ongoing throughout the life cycle. Hence, in this respect, development is not solely about what happens in the first few years. It occurs throughout a lifetime, interacting with what organisms encounter along the way. As such, understanding life means understanding the many different paths that life takes based on the obstacles and changes it faces. It is never solely about understanding the genetic code. In this article, we advocate this sense of development as the ongoing action and reaction of organisms during their life.

### Static, dynamic, and developmental views with Kanner and Asperger

The concept of development and genes also play a role in autism's history—a history that is wellknown and amply documented (63, 64). Leo Kanner, a psychiatrist of Austrian descent, is the person most associated with establishing the concept of autism. He founded the department of child psychiatry at the John Hopkins Hospital in Baltimore in the 1930s. In 1943, he wrote the seminal text “Autistic Disturbances of Affective Contact” (65, 66). Readers of this paper are advised to read this original text if they have not done so already. It is often assumed that the children Kanner described all exhibited features of what we would now call “Kanner's” autism, unlike the children his German counterpart, Hans Asperger, described. Nevertheless, the text describes various children, all with their own challenges and personalities. In the text, Kanner suggests that autistic children, unlike children with childhood schizophrenia, do not withdraw from the world but are born with the condition (1). He also describes how the children gradually come out of their shelves toward the world: “our children gradually compromise by extending cautious feelers into a world in which they have been total strangers from the beginning” (1, 66). In later texts, he describes the adults some of the children have become: how many of them had gradually acquired social skills and how many had succeeded in finishing their education and establishing a place in society (67). So, these texts show that, although Kanner stressed that autism is innate, it is not a static, unchanging given. Kanner firmly describes autism as a developmental phenomenon: not solely because its symptoms become apparent in the first developmental years but also because its manifestation changes throughout the life course. This approach contrasts with de descriptions of Hans Asperger, the German pediatrician. He gave his name to the wellknown Asperger syndrome, which was until recently considered a subtype of autism. It is often thought that Leo Kanner described more pronounced cases of autism, whereas Hans Asperger focused on autistic children without intellectual disability, what he called “little professors.”^7^ However, even in his seminal text “Die ‘Autistische Psychopathen' im Kindesalter,” not all children are intellectually gifted, and it contains descriptions of children with various behaviors (70). We believe the major differences between the two texts do not lie in the kind of behaviors children exhibit but rather in how the authors appreciate autism. As stated before, Kanner, a child psychiatrist, stressed the innateness of autism but also as developmental, dynamic, and adaptive to circumstances. After all, Leo Kanner wanted to jumpstart the field of child psychiatry in the United States. Such a description would probably serve better for that aim than a description that suggests autism is a static psychopathology. Asperger, however, saw autism as a personality disorder, a more static trait of one's personality that one is born with and with which one dies. We argue that the ideas that these archfathers of autism had about its nature reflect the different appreciations of autism today. On the one hand, autism is a developmental condition, of which the course is not fixed, and on the other hand autism is an innate neurological “difference” with strengths and weaknesses.

### Static, dynamic, and developmental views today

The term developmental in developmental disorder can have different meanings. For instance, development in the context of *developmental disorder* can refer to the idea that the symptoms of a disorder are present early in life, in what is considered the developmental period. As such, the term developmental disorder is compatible with a view that sees autism as primarily static, genetic and innate. However, in biology and philosophy, development instead emphasizes dynamics (61, 62). For instance, a developmental theory of life stresses that what an organism is and how it functions is not only the result of genetic makeup or influences *in utero* or very early in life. From birth to death, organisms are in development: they maintain themselves and adapt in response to the specific contexts (physical, psychological, social, and cultural) they find themselves in. In this view, behavior is not solely the result of one's genetic programming but a meaningful response to what happens around us. This ties in with recent findings regarding systems biology and developmental systems thinking (58, 71). In thinking about organisms, genes have been losing their prime position as the final explanation of behavior and form. Such approaches also imply that looking at individual cases and situated experiences next to statistical tendencies in development is crucial. Systems biology seems to tell us that if we want to understand life, we need to understand both specific lives and life in general. We will come back to that later on.

Although systems biology approaches are gradually finding their way into autism research (3), and epigenetic effects and other omics studies become increasingly prevalent (72), most autism research can still be subdivided into two strands. A first strand of autism research is the already mentioned fundamental genetic, neurological and psychological research into the “causes” of autism. We have discussed the reasons and implications of the search for autism explanations above. The rationale of this kind of research mainly ties in with the view of autism as innate, fixed, and related to how our genes and brains work, although, as we also already stated, most researchers acknowledge that the reality of autism's biology is much more complex (20, 21). A second strand of autism research is research into early detection and intervention (73). This strand is not wholly separate from the search for causal explanations in the sense that there lingers hope that finding suitable biomarkers will aid the discovery of autism even before autistic behavior is present in young children (74). This is thought to have several benefits: parents will be more prepared to tackle specific challenges their child may face and understand their child better. It is also often claimed that early detection will enable early intervention. The idea that autism can be “prevented” through early intervention is heavily contested, as autistic people have asserted their rights to exist as autistic people (50, 75).

Hence, researchers into early intervention must balance a tight rope of advocating benefits for autistic people early on but not claiming that what they are targeting is autism traits. The assumption behind early intervention is that there is a critical developmental period in which brains are still flexible enough to be influenced, as neuronal plasticity is greatly enhanced in that period (76). We do not want to question the idea that brain plasticity is highest during the earliest developmental period, and we do not challenge the importance of proper care during this period. Nevertheless, we want to suggest a more encompassing view of “development.” Indeed, current biological knowledge demonstrates that development is *ongoing throughout life* (77–79). This means that early childhood experiences, although relevant and crucial, do not necessarily set a person's further life course in stone. Speaking with the words of neuroscientist Francisco Varela, quoting a verse by the poet Antonio Machado, life is “laying down a path in walking” (80). The path does not stop after the first 3 years. Indeed, we believe that dynamic models of life and mind offer possibilities to look at neurodevelopmental conditions differently. Rather than seeing autistic people as people with fundamental flaws in their genes or software deficiencies in their brains that have to be explained in reductionist terms, autism appears as a phenomenon that exists in interaction with the context, as a meaningful reaction to the environment. Taking a developmental diversity approach in research will give credit to this reality.

## Whereto, autism research?

What does it mean to study developmental diversity rather than “autism”? What would taking a developmental diversity approach to research into childhood disability mean? We acknowledge that researchers already accept that studying such a diverse collection of experiences and biologies covered by the term “autism” is nearly impossible. It has been suggested that approaches such as the Research Domain Criteria may help look at autism and its causes more granularly (81). Moreover, the WHO's ICF framework has been used to develop core sets for autism that allow studying autism beyond the medical model in terms of functioning (82). A developmental diversity approach could integrate these approaches and take a step further by incorporating methods and insights from the humanities.

First, we argue that research, when taking a developmental approach, could take temporality into account, as it is crucial to incorporate dynamics and changes. The notion of “crip time” from Disability Studies can function as a way of thinking about such dynamics (83). Second, we highlight the importance of incorporating experience and understanding in studying developmental diversity. Therefore, such research is equally sensitive to general tendencies and quantitative measures of individual experiences and qualitative information. Third, we argue that a developmental diversity approach does not stop at disciplinary or diagnostic boundaries. It involves engaging with people from different neurotypes as co-creators of the research and encouraging fruitful collaboration between different disciplines, from genetics to psychology to the humanities and philosophy.

### The role of longitudinal research and appreciating temporality

Appreciating development, as described above, as the lifelong dynamics of organisms interacting with the environment, has implications for autism research. For one, it may mean that research should put less emphasis on searching for explanations (“the hunt for genes”) and more on investigating systemic biological and psychological processes and how they change or remain the same throughout a lifetime. With this, we do not want to suggest that research into genes is worthless. It could be the starting point for a more systemic approach that looks at organisms and people as the complex systems they are (84). Granted, many autism researchers we have spoken to already dream of such research and acknowledge the importance of longitudinal research to study the interaction between genes and environment and the factors that can help increase quality of life. At the same time, the way research practices are set up nowadays makes such longitudinal research almost impossible. In the timeframe of a typical 4-year research project, finding a genetic variant associated with a specific family may be possible, and this is a good outcome for a PhD. However, it is nearly impossible to investigate what this variant means at different stages in life and how it interacts with other factors if there is no guaranteed long-term funding. Moreover, many topical funding calls still use categorical diagnostic categories, forcing researchers to formulate their research plan in terms of these categories, as if they were fixed and stable entities to be grasped. Systemic and developmental approaches to autism research require systemic changes to research funding.

At the same time, we believe that when studying the diverse paths that development can take, an appreciation of diversity is also essential. It would be tempting to revert to research about “normal” vs. “abnormal” development. However, in our view, a developmental diversity approach challenges the concept of normal development. Researchers of developmental diversity could be inspired by the concept of “crip time,” a term from disability studies. We will briefly elaborate on the concept as Alison Kafer and others conceived it (83).

Disability, as Alison Kafer demonstrates, is very often described in relation to time (i.e., prognosis, developmental disorder, chronic, childhood disability, medical history, etc.). These temporal framings are animated by a “curative imaginary,” leading Kafer to the concept of “curative time.” Curative time is a way to conceive disability in relation to normative temporalities (i.e., a linear understanding of a “future perfect,” “a developmental correctness,” and “the window of opportunity”). This curative imaginary is omnipresent in clinical programs in early childhood (83). Detecting early autism characteristics comes down to noticing whether the child develops the right skills at the right moment in time, compared to the “normal” temporal schedule of development. Early interventions are acclaimed to offer better odds of living well in the future when provided at the right time during the right window of opportunity. Most autism researchers know that the ambition to “cure” autism is long past its expiration date.

Nevertheless, it is still a challenge in the early intervention literature, if not impossible, to imagine a flourishing future for autistic children, at least not without deploying clinical interventions and without straightening the developmental path (50, 85). The idea is that an autism developmental path without interventions is a path no one wants. Kafer's aim is to challenge this, as the futures we envisage can reveal the biases of the present. Kafer, therefore, disrupts the linear, progressive, modernist, directional, getting better marking of time and development. For this purpose, Kafer conceptualized the idea of “crip futurities.” Crip futures incorporate multiple, shifting, affective understandings of temporality that make space for, imagine and enact futures that include the bodyminds left out of normative renderings of personhood and futurity (28). Research could, for instance, speculate with parents and healthcare professionals on an autistic child's future beyond curative imaginary (33).^8^ It implies that studying developmental diversity is also studying neurodivergent flourishing and investigating which environment can enable that flourishing.

### The role of experience

A developmental perspective on life implies that the study of said life should pay equal importance to general statistical and quantitative tendencies as to individual trajectories and experiences. General trends are not more scientific than research into specific cases. They both shed a different light on reality. However, until recently, quantitive and generalizable abstract data were considered far more scientific and even “real” than cases. However, given the partial open-endedness of development, from a developmental perspective, specific cases and life trajectories yield equally exciting and essential information. The study of such life trajectories should include the study of experiences (such as narratives and other creative forms of expressing experiences) in biological research and extend beyond genes-environment dichotomies. In our view, autistic behavior has substance; it is not the result of an infection or mutated genes but a *meaningful response to context and biology* (16). To understand this meaningful response, biological research needs to be complemented by how specific behavior is related to a particular experience of the world; explaining and understanding must go together (15, 50, 86).

Such an approach allows us to build another bridge between the life sciences and the humanities. After all, there is already a pile of humanities research that argues in favor of looking at autism more ambiguously and incorporating experience stories (2, 18, 87–90). Autistic experiences change throughout one's life and what autism means has to be actively integrated into one's own multi-facet story repeatedly. In our research, we have experienced that a purely explanatory approach to autistic people does no justice to the experiences of these people in interaction with their environment.

A developmental diversity approach is neurodiversity-affirmative research and thus can also pay much more attention to the autistic experience by acknowledging the heterogeneity and indeterminacy inherent in developmental conditions such as autism. This indeterminacy, moreover, has two forms (2). On the one hand, there is interpersonal indeterminacy which means that there are fundamental differences among autistic people. On the other hand, there is intra-personal indeterminacy which means that even for the person facing a number of challenges at some point in their lives, it makes no sense to view these challenges as only the results of genes. Those challenges always depend on the specific context and previous experiences of the particular person (2). Consequently, autism may have different meanings depending on the life stage and context of the individual. Hence, understanding lived experiences is also indispensable.

For instance, in their phenomenological research on the experiences of adults who got their autism diagnosis later in life, Hens and Langenberg focused on how a formal clinical diagnosis changed autistic people's relationships with others and themselves. Some participants recognized themselves immediately in their diagnosis, while others needed more time to explore what the diagnosis could mean and do for them. For instance, Karel, 55 years and diagnosed when he was 40, said the following:

*It offers an insight that can inspire, that can help you reorient yourself. But you still have to make it your own so that you can build it into your own actions. For example, now I can accept that I may sometimes go into too much detail. But that is again simplifying it. A diagnosis offers focal points, which you can research. How does this fit into my own pattern of actions? It is an extra critical factor that can be confronting or can offer peace of mind and a way to think about it. That was not explained to me when I received my diagnosis because the world of diagnoses is hyper flat* *(**70**)*.

This and other conversations with and stories from autistic adults exemplify that (however much they have experienced problems and have felt different from others) their experiences cannot be easily be categorized or pinned down. Instead, we notice how a, perhaps neurological, vulnerability can lead to dysfunction at a certain point in life and, at the same time, how people have dealt with such vulnerability in their interactions with others throughout their lives. Probably, there is a certain predisposition—genetic or congenital—to atypical cognitive or social development that is not always “translated” into dysfunction. Moreover, it is particularly enlightening to notice how these people have dealt with their challenges before and after their diagnosis and how they learn from this. This suggests that an approach and research that focuses exclusively on problems, difficulties and causes in the individual (as embedded in the dominant autism discourse) is problematic and often beside the point. Research methodologically oriented toward lived experiences and how people interpret and narrate their own experiences allows for assessing the suitability of specific explanatory models. Moreover, we believe it is of utmost importance that research participants are not only enrolled as subjects whose experiences can be queried and investigated. Instead, people from different neurotypes should be actively engaged in co-creating relevant and meaningful research. Ensuring an ethical scientific and clinical practice entails including the viewpoints and explicitly paying attention to those who have held marginalized positions in healthcare. If we want to understand what health and pathology mean for different people, this means engaging honestly with those who have been ignored.

### Crossing disciplinary and diagnostic boundaries

A substantial number of people diagnosed with autism have additional diagnoses such as ADHD, dyslexia, dyspraxia or intellectual disability. In some cases, autism is associated with a specific syndrome, such as Fragile X (91). Like the concept of development itself, the concept of comorbidity is equally hard to grasp. Does it mean that a specific neurodivergent person, who has a diagnosis of autism and ADHD, has two separate conditions that happen to occur in the same person? Is Fragile X the cause of autism? Or is the concurrence of, for example, autism and dyslexia a symptom of an underlying neurotype that can explain both, such as enhanced perceptual functioning? As present-day research often starts from diagnostic categories, it is unlikely to shed light on this matter (92). However, genetic research has indeed suggested that there is more overlap between the different categories than a categorical approach would suggest. An approach that would embrace the idea of developmental diversity could shed some valuable light on such comorbidities. We believe that such an approach could be most successful if development is studied as such, without starting from categories and including children and people who may not receive a diagnosis but may be diverse in their own ways.

Besides transcending diagnostic categories, research that wants to study life in all its diversity and that appreciates individual experiences as of utmost importance to understanding life presupposes an interdisciplinary approach. Such an approach includes vital input from social sciences, humanities and arts-based research and foregrounds complexity, ambiguity, and multiple socialities as the baseline of (autism) research. Indeed, we believe there is no need for a hierarchy between the exact sciences and the humanities regarding understanding development. Scholars in the humanities can join research consortia, not to serve exact scientists to write the informed consent forms for them but to provide a different kind of insight into studying the phenomenon at hand. It is equally important to include neurodivergent researchers in the research projects. In the words of Jorn Bettin:

“*Neurodiversity friendly forms of collaboration hold the potential to transform pathologically competitive and toxic teams and cultures into highly collaborative teams and larger cultural units that work together more like an organism rather than like a group of fighters in an arena”* *(**93**)*.

Finally, we also want to mention *neurodiversity studies* here, a new field of inquiry that aims to find new ways to support including neurodivergent perspectives in knowledge production. It questions the theoretical assumptions surrounding idea of the neurotypical (39). It analyses the role of neuronormativity in theory and science and aims to contribute to redefining what it means to be human (39, 94–97). We believe that any autism or developmental disability project should engage with fields such as neurodiversity studies or disability studies.

## Some afterthoughts

In this paper, we have proposed developmental diversity as a concept that can function as a framework for neurodiversity sensitive approach. We have explored what a research practice that starts from developmental diversity could entail. We hypothesize that thinking with developmental diversity rather than categorical difference represents an opportunity for a more inclusive society and fundamentally can alter how we perform research. As such, it is in line with requests of neurodiversity and disability movements. Such an approach appreciates the temporalities and dynamics of experience and focuses on flourishing for all types of people. We did not give specific suggestions on how such an approach could be implemented in terms of methodological tools. As philosophers and humanities scholars, we do not have the expertise to suggest the variables sensitive to the dynamics of experience and temporality that should be included in the databases or what kind of statistics that could be used to include individual experiences. We hope people more knowledgeable in experimental psychology will take up the challenge. We also acknowledge that, at the moment, our proposed research may seem utopian. For one, although almost all autism researchers we speak with are sympathetic to such an approach and appreciate the need for longitudinal research into flourishing and away from diagnosis-based approaches, it remains the case that existing resources such as databases often are still based on such diagnostic categories. Moreover, funders often focus on specific categories as well, and particularly autism as a category is a phenomenon that seems to be of great interest to funding agencies.

Furthermore, it is often helpful for people to think about themselves in terms of autism, ADHD, or another neurodivergent identity. However, our suggestion is not to abandon these identities or to suggest that they are not real or mere constructs. They denote real experiences and are a valuable means of communication with those with similar experiences. At the same time, studying developmental diversity and flourishing over a lifetime of many neurotypes may very well be an approach that is acceptable to the neurodivergent community. Whether that will be the case remains to be seen. Research practices and ideas about development may not change over time and will require a gradual shift in research discourse. With this paper, we hope to have contributed our drop in the ocean to enable such a shift.

## Author contributions

All authors listed have made a substantial, direct, and intellectual contribution to the work and approved it for publication.

## Footnotes

^1^On the basis of interviews with scientists, Hollin connects the heterogeneity of autism to uncertainty and states that we need to reflect more on the concept of uncertainty in autism research as uncertainty can mean many things, e.g., epistemic uncertainty and ontological uncertainty (2).

^2^The DSM is the Diagnostic and Statistical Manual of Mental Disorders which provides the standard language by which clinicians, public health officials and researchers in the United States and most European countries as well communicate about mental disorders. The current edition is the fifth edition (DSM-5) and was published in 2013 (5). There are of course also other classification systems, like the one of the World Health Organization (WHO), which is the International Classification of Diseases (ICD) (6).

^3^In general more participatory action-research with adults and children with substantial intellectual, language and behavioral challenges is needed. As Tesfaye et al. and Van Goidsenhoven et al. rightly point out, this group is often neglected in research—also in more traditional autism research (35, 36). It can be argued that we need to be much more invested and creative in exploring the experiences of this group of people (and thus also collect data about their temporality and dynamics of experience). Research that does this, is mostly qualitative oriented and integrates arts-based research methods (33, 37, 38).

^4^For a clear introduction on how the neurodiversity movement is often misunderstood and how this paradigm is certainly welcoming (neurodiversity-affirmative) therapy, intervention, and support see: den Houting (23).

^5^Examples of this impact are pointed out by Leadbitter et al. (31) and include: (a) debates over whether the social difficulties experienced by autistic people are best understood as being a problem within the individual, or a problem between two (mis-matched) individuals, and the resulting research into the Double Empathy Problem and diversity in social intelligence. (b) On improving mental health and quality of life in autistic individuals and an increase in research into effective, person-centered mental health interventions. (c) Research into community preferences over the language used to describe autism and autistic people.

^6^Drawing on both theoretical and empirical research, Chapman (24) argues hat the ecological model has greater utility for research and practice than the leading and dominant psychiatric functional analysis of mental functioning. The ecological model, however, is not used as a rival to evolutional models *per se*: Chapman's ecological model is focused on understanding human mental functioning only, and not for understanding biological functioning.

^7^During the last two decades, several studies appeared with interesting analyses of how such metaphors as “little professors” in medical texts has influenced (and still influences) autism representations in popular culture and discourse (68, 69).

^8^Leni Van Goidsenhoven and Elisabeth De Schauwer did this kind of research in co-creation with a non-verbal young woman (33).
